# Dementia knowledge, risk reduction awareness, misconceptions, and stigma in Kuwait: a cross-sectional study

**DOI:** 10.3389/fpubh.2026.1941519

**Published:** 2026-10-19

**Authors:** Abdelmoneim Awad, Aseel Almutairi

**Affiliations:** Department of Pharmacy Practice, College of Pharmacy, Kuwait University, Safat, Kuwait

**Keywords:** dementia knowledge, dementia prevention, Kuwait, misconceptions, modifiable risk factors, public awareness, stigma

## Abstract

**Background:**

Dementia represents a growing public health challenge globally, particularly within aging populations. Although no cure currently exists, evidence suggests that targeting modifiable risk factors can substantially lower dementia risk. However, limited public knowledge, widespread misconceptions, and stigma remain significant barriers to early diagnosis, preventive action, and timely care. This study aimed to assess general dementia knowledge, awareness of risk and protective factors, misconceptions about risk reduction, and stigma toward people living with dementia (PwD) among the general population in Kuwait. It also sought to identify predictors of knowledge and stigma.

**Methods:**

A quantitative cross-sectional study was conducted among 733 adults residing in Kuwait. Participants completed a validated, self-administered questionnaire distributed through social media and email using a snowball sampling approach. The survey incorporated the Dementia Knowledge Assessment Scale (DKAS) for general knowledge, the Knowledge of Dementia Risk Reduction Scales (KoDeRR-R and KoDeRR-M) for awareness of modifiable risk factors and misconceptions, and the Dementia Public Stigma Scale (DePSS) for stigma-related issues. Descriptive statistics, correlation analyses, and logistic regression were used to analyze the data.

**Results:**

Overall dementia knowledge was low, with a median DKAS score of 19 out of 50. Medical professionals and students (AOR = 8.53, 95% CI: 5.11–14.23) and those with professional experience working with PwD (AOR = 2.45, 95% CI: 1.36–4.42) exhibited significantly higher knowledge. Conversely, married participants scored lower (AOR = 0.47, 95% CI: 0.26–0.85). Respondents showed low awareness of modifiable risk factors (median KoDeRR-R = 16/28) and struggled to identify misconceptions (median KoDeRR-M = 0/12). The median number of reported risk-reducing behaviors was 7 out of a possible 14. Stigma levels were moderate (median DePSS = 62/112) and were significantly lower among those with professional experience working with PwD (AOR = 2.04, 95% CI: 1.36–3.07). Higher dementia knowledge correlated significantly with greater risk factor awareness, more engagement in preventive behaviors, and lower stigma (*p* < 0.05).

**Conclusion:**

This study highlights critical gaps that underscore the urgent need for comprehensive public health initiatives to improve dementia literacy, correct misconceptions, promote preventive actions, and reduce stigma across the population.

## 1 Introduction

Dementia refers to a group of progressive neurodegenerative disorders that impair cognitive and functional abilities (1, 2). Although it predominantly affects older adults, it is not an inevitable consequence of aging (2) and creates significant emotional, social, and economic burdens globally (2, 3). While Alzheimer’s disease (AD) is the most prevalent form, various subtypes and overlapping etiologies exist, occasionally emerging as young-onset cases (4–6). Beyond cognitive decline, people living with dementia (PwD) experience a range of progressive neuropsychiatric and physical challenges that lead to progressive loss of independence (2, 4).

As the seventh leading cause of death from noncommunicable diseases (2) dementia affected over 55 million people globally in 2020, with prevalence and economic costs projected to rise sharply, particularly in low- and middle-income countries (7). Despite this growing burden, the condition remains widely underdiagnosed (8). In the Arab world, dementia affects an estimated 1.3 million people, generating substantial economic costs (9). While Kuwait reports comparatively lower prevalence rates (1.19% for adults aged ≥50 years and 2.99% for those aged ≥60 years) (9), these figures still highlight a clear need for focused public awareness and prevention efforts.

Improving public knowledge of dementia symptoms, risk factors, and management is vital for promoting early detection, facilitating timely care, and reducing social stigma (10, 11). Family members are often the first to notice early symptoms and help patient’s access medical care. Because of this, national strategies increasingly emphasize public education as a cornerstone for improving patient outcomes (12). An informed public is better equipped to challenge misconceptions, reduce stigma, and adopt evidence-based preventive behaviors (13). However, widespread misconceptions remain common. Many people still mistakenly believe dementia is a normal, unpreventable part of getting older (14, 15). Knowledge regarding risk reduction is low; the public often misunderstands how cardiovascular health affects the brain, and many endorse unproven strategies to prevent cognitive decline (14–16).

There is no cure for dementia, but evidence suggests that modifiable risk factors account for approximately 45% of cases in high-income countries, and likely more in low- and middle-income countries. These factors include early-life education deficits, hearing loss, traumatic brain injury, hypertension, obesity, smoking, alcohol misuse, diabetes, depression, physical inactivity, social isolation, air pollution, untreated vision loss, and high LDL cholesterol (17). The World Health Organization has made dementia risk reduction a global priority. Yet, for such strategies to be effective, greater public awareness and engagement are essential (18). Beyond prevention, the stigma attached to dementia severely impacts the well-being of both PwD and their caregivers, leading to social exclusion, psychological distress, caregivers’ burden, and heavy emotional strain (19, 20). When low awareness combines with fear, people are less likely to seek timely diagnosis and treatment (21). Consequently, many countries have made stigma reduction a core component of their national action plans (22).

Recent literature confirms that low dementia knowledge and limited awareness of risk reduction strategies are global issues. An Australian national survey revealed a poor understanding of dementia and its modifiable risks, and widespread misconceptions, though stigma toward dementia and PwD was relatively low (23). Similar knowledge gaps exist in Europe; studies from Ireland and Norway noted that while people recognize risks like head injury or low cognitive stimulation, they often overlook factors like hearing loss, diabetes, and depression (24, 25). A study conducted in Poland found that public knowledge about dementia and its risk factors is insufficient (26). Research from Germany showed reasonable public awareness of physical and cognitive activities as key protective factors, as well as recognition of social isolation as a risk factor for dementia. However, knowledge of the contribution of cardiovascular risk factors to dementia was found to be low to moderate (27). In Switzerland and northern Italy, misconceptions and limited knowledge about causes, risk factors, and care strategies remain prevalent (28).

In the Middle East, data on public knowledge of dementia is sparse. A recent Saudi Arabian study using the latest version of the Dementia Knowledge Assessment Scale (DKAS) reported insufficient knowledge of dementia, particularly regarding risk factors and communication with PwD, with only half of participants recognizing the role of healthy lifestyle choices in dementia prevention (29). Other Saudi studies have focused specifically on Alzheimer’s disease, assessing awareness, knowledge, and attitudes among the general public and healthcare students (30–34). In Kuwait, research has mostly targeted specific subgroups rather than the general population. A 2019 study among university students using an earlier version of the DKAS found moderate levels of dementia knowledge (35). More recently, a 2023 study among healthcare professionals revealed low knowledge levels, particularly among non-physician professionals (36).

Given the urgent global and regional need for dementia prevention and despite the Kuwaiti government’s growing emphasis on healthy aging, there remains a lack of comprehensive studies examining the public knowledge of dementia and its modifiable risk factors, and stigma toward PwD. This gap is concerning, given that misconceptions, limited awareness of risk-reduction strategies, and stigma may impede early diagnosis, limit preventive behaviors, and undermine effective care. Therefore, this study aimed to assess general dementia knowledge, awareness of risk and protective factors, misconceptions about risk reduction, and stigma toward PwD among the general population in Kuwait. It also sought to identify predictors of general dementia knowledge, knowledge of risk factors and misconceptions, and stigma toward PwD. Ultimately, these findings will provide the necessary evidence to design targeted, culturally appropriate public health campaigns that improve dementia literacy, encourage proactive risk reduction, and reduce social stigma.

## 2 Materials and methods

### 2.1 Study design and population

A descriptive, cross-sectional survey was conducted targeting the general population of Kuwait aged 18 years and older. Located in the Middle East, Kuwait had an estimated population of 4.88 million in 2025 (37). While the overall population is heavily structured by an expatriate workforce, Kuwaiti nationals comprise approximately 1.57 million individuals. Within this national demographic, females slightly outnumber males (approx. 793,000 vs. 773,000), and the adult Kuwaiti population (aged 18 and older) is estimated at approximately 1 million (37). The Medical Research Ethics Committee of the Kuwait Ministry of Health reviewed and approved this study (Reference No. 2024/108).

### 2.2 Sample size and sampling strategy

The sample size was determined using the PS Power and Sample Size Calculator version 3.1.2 (38). Based on the primary outcome of general dementia knowledge, a minimum of 618 participants was required to detect a conservative 20% difference in proportions between major demographic groups (e.g., gender or educational levels) with 80% power and a 5% significance level. A 20% difference was selected as a practically meaningful effect size for public health awareness surveys where baseline population proportions are previously undocumented. Respondents were recruited using a non-probability online snowball sampling strategy.

### 2.3 Data collection

The survey link was distributed via social media platforms (WhatsApp, X, Instagram, and Telegram) and email. The invitation briefly outlined the study objectives, emphasized that participation was voluntary, and assured confidentiality and anonymity. Before accessing the questionnaire, participants had to read and electronically accept an informed consent form. To increase the reach and diversity of the sample, participants were encouraged to share the survey within their social networks.

In total, 733 individuals completed the survey (719 in Arabic, 14 in English). The survey was completed anonymously and took approximately 15–20 min to finish. To ensure data completeness, the electronic survey was designed to require responses to all questions before submission, resulting in a complete dataset.

### 2.4 Study questionnaire

Permission was obtained from the Australian Institute of Health and Welfare to adapt the validated English and Arabic versions of the Dementia Awareness Survey (23). The sociodemographic section was modified to fit the local Kuwaiti context. Both versions were then pretested with 10 individuals (adults representing the general target population) to assess readability and clarity; data from these pretest participants were excluded from the final analysis. After incorporating minor feedback regarding formatting and specific wording from the pretest, the final questionnaire consisted of six domains:

1. *Demographic and other characteristics*: 14 items capturing sociodemographic and other characteristics data, such as age, gender, nationality, educational level, employment status, and health-related information (Table 1).
2. *General dementia knowledge*: The 25-item Dementia Knowledge Assessment Scale (DKAS) (39) evaluated understanding across four subdomains: causes and characteristics (7 items), communication and behavior (6 items), care considerations (6 items), and risks and health promotion (6 items) (Table 2). Participants responded using a modified 5-point Likert scale (True, Probably True, Probably False, False, or Do Not Know).
3. *Knowledge of dementia risk reduction*: The Knowledge of Dementia Risk Reduction (KoDeRR) scale (40) was used to assess awareness of 14 evidence-based modifiable risk factors and 6 common misconceptions (Table 3). Responses were recorded on a 5-point Likert scale (1 = Strongly Disagree to 5 = Strongly Agree).
4. *Actions or behaviors for dementia risk reduction*: 14 items assessed whether participants actively engaged in actions or behaviors that might reduce dementia risk (Table 4). For each behavior, participants indicated if they performed it specifically to reduce dementia risk, for other reasons, or not at all, along with a “Prefer not to say” option.
5. *Stigma toward PwD*: The Dementia Public Stigma Scale (DePSS) (41) assessed public stigma toward dementia across three dimensions: cognitive (dementia-related stereotypes; 10 items), emotional (negative prejudices and emotional reactions; 4 items), and behavioral (discriminatory behaviors; 2 items), as presented in Table 5 of the Results section. Respondents rated their level of agreement on a 7-point Likert scale (1 = Strongly Disagree, 2 = Disagree, 3 = Somewhat Disagree, 4 = Neither Agree nor Disagree, 5 = Somewhat Agree, 6 = Agree, 7 = Strongly Agree).
6. *Help-seeking and information-seeking behaviors*: Six items explored participants’ willingness to seek medical or professional support, as well as their preferred sources and frequency of accessing dementia-related information.

**Table 1:** Sociodemographic and other characteristics of the respondents (n = 733).

Characteristics	Frequency (%)
Age (years)	
18–24	264 (36.0)
25–34	151 (20.6)
35–44	151 (20.6)
≥45	167 (22.8)
Gender	
Male	183 (25.0)
Female	550 (75.0)
Nationality	
Kuwaiti	640 (87.3)
Non-Kuwaiti	93 (12.7)
Marital status	
Single	354 (48.3)
Married	341 (46.5)
Divorced	26 (3.6)
Widowed	12 (1.6)
Educational level	
Less than high school	8 (1.1)
High school	46 (6.3)
Diploma	60 (8.2)
University undergraduate	242 (33.0)
University graduate	314 (42.8)
Postgraduate	63 (8.6)
Employment	
Unemployed	29 (3.9)
Retired	79 (10.8)
Housewife	8 (1.1)
Student	265 (36.2)
Professional	90 (12.3)
Self-employed	11 (1.5)
Clerical	251 (34.2)
Residence area	
Capital	136 (18.6)
Hawalli	100 (13.6)
Al-Farwaniyah	215 (29.3)
Al-Ahmadi	122 (16.6)
Al-Jahra	76 (10.4)
Mubarak Al-Kabeer	84 (11.5)
Monthly average income	
Less than 500 KD	270 (36.8)
500–1,000 KD	163 (22.2)
Greater than 1,000 KD	300 (40.9)
Working or studying in the medical field	
No	619 (84.4)
Yes	114 (15.6)
If yes, working or studying in the medical field (*n* = 114)	
Medical doctor	12 (10.5)
Dentist	3 (2.6)
Pharmacist	24 (21.1)
Allied health specialist	19 (16.7)
Nurse	1 (0.9)
Medical student	5 (4.4)
Dental student	2 (1.8)
Pharmacy student	42 (36.8)
Allied health student	4 (3.5)
Nursing Student	2 (1.8)
Have been diagnosed with dementia	
No	727 (99.2)
Yes	6 (0.8)
Have known a family member and/or a friend with dementia	
No	380 (51.8)
Yes	353 (48.2)
Have known (*n* = 353) and cared for a family member and/or a friend with dementia	
No	176 (49.9)
Yes	177 (50.1)
Have worked directly with people living with dementia	
No	582 (79.4)
Yes, currently work with people with dementia	62 (8.5)
Yes, used to work with people with dementia	89 (12.1)

**Table 2:** General dementia knowledge (*n* = 733).

Statements about dementia	False for an untrue statement/ True for a true statement *n* (%)	Probably False for an untrue statement/ Probably True for a true statement *n* (%)	Do not know or wrong answer *n* (%)	Mean (SD) out of a maximum score of 2	Median (IQR) out of a maximum score of 2
Causes and characteristics					
Dementia is a normal part of the aging process ^	129 (17.6)	128 (17.5)	476 (64.9)	0.53 (0.77)	0 (1)
People can recover from the most common forms of dementia ^	126 (17.2)	218 (29.7)	389 (53.1)	0.64 (0.75)	0 (1)
Dementia does not result from physical changes in the brain ^	285 (38.9)	144 (19.6)	304 (41.5)	0.97 (0.89)	1 (2)
Planning for end-of-life care is generally not necessary following a diagnosis of dementia^	328 (44.7)	95 (13.0)	310 (42.3)	1.02 (0.93)	1 (2)
Vascular dementia (blood vessel disease) is the most common form of dementia ^	52 (7.1)	59 (8.0)	622 (84.9)	0.22 (0.56)	0 (0)
Most forms of dementia do not generally shorten a person’s life ^	94 (12.8)	130 (17.7)	509 (69.4)	0.43 (0.70)	0 (1)
Alzheimer’s disease is the most common form of dementia *	379 (51.7)	230 (31.4)	124 (16.9)	1.35 (0.75)	2 (1)
Risks and health promotion					
Maintaining a healthy lifestyle does not reduce the risk of developing the most common forms of dementia^	244 (33.3)	152 (20.7)	337 (46.0)	0.87 (0.88)	1 (2)
Early diagnosis of dementia does not generally improve quality of life for people experiencing the condition^	291 (39.7)	164 (22.4)	278 (37.9)	1.02 (0.88)	1 (2)
The sudden onset of cognitive problems is characteristic of common forms of dementia^	44 (6.0)	49 (6.7)	640 (87.3)	0.19 (0.52)	0 (0)
Symptoms of depression can be mistaken for symptoms of dementia*	89 (12.1)	165 (22.5)	479 (65.3)	0.47 (0.70)	0 (1)
Having high blood pressure increases a person’s risk of developing dementia*	92 (12.6)	154 (21.0)	487 (66.4)	0.46 (0.70)	0 (1)
Exercise is generally beneficial for people experiencing dementia*	246 (33.6)	272 (37.1)	215 (29.3)	1.04 (0.79)	1 (2)
Communication and behavior					
It is impossible to communicate with a person who has advanced dementia ^	157 (21.4)	169 (23.1)	407 (55.5)	0.66 (0.80)	0 (1)
A person experiencing advanced dementia will not generally respond to changes in their physical environment ^	68 (9.3)	79 (10.8)	586 (79.9)	0.29 (0.62)	0 (0)
It is important to correct a person with dementia when they are confused ^	138 (18.8)	117 (16.0)	478 (65.2)	0.54 (0.79)	0 (1)
Medications are the most effective way of treating behavioral symptoms of dementia^	54 (7.4)	101 (13.8)	578 (78.9)	0.29 (0.59)	0 (0)
People experiencing advanced dementia often communicate through body language *	148 (20.2)	200 (27.3)	385 (52.5)	0.68 (0.79)	0 (1)
Uncharacteristic behaviors in a person experiencing dementia are generally a response to unmet needs *	142 (19.4)	229 (31.2)	362 (49.4)	0.70 (0.77)	1 (1)
Care considerations					
People experiencing dementia do not generally have problems making decisions^	351 (47.9)	102 (13.9)	280 (38.2)	1.10 (0.92)	1 (2)
Movement is generally affected in the later stages of dementia*	237 (32.3)	219 (29.9)	277 (37.8)	0.95 (0.83)	1 (2)
People with advanced dementia may have difficulty speaking*	253 (34.5)	219 (29.9)	261 (35.6)	0.99 (0.83)	1 (2)
People experiencing dementia often have difficulty learning new skills*	330 (45.0)	191 (26.1)	212 (28.9)	1.16 (0.84)	1 (2)
Difficulty eating and drinking generally occurs in the later stages of dementia*	258 (35.2)	209 (28.5)	266 (36.3)	0.99 (0.84)	1 (2)
Daily care for a person with advanced dementia is effective when it focuses on providing comfort*	302 (41.2)	219 (29.9)	212 (28.9)	1.12 (0.82)	1 (2)

*True Statement ^False Statement.

**Table 3:** Knowledge of dementia risk reduction (*n* = 733).

Evidence-based Modifiable risk factors	Strongly agree *n* (%)	Agree *n* (%)	Strongly disagree/disagree/neutral *n* (%)	Mean (SD) out of a maximum score of 2	Median (IQR) out of a maximum score of 2
Doing regular physical activity, such as playing sport, brisk walking or gardening	177 (24.1)	390 (53.2)	166 (22.6)	1.02 (0.68)	1 (0)
Continually learning new things (such as languages, music, hobbies)	160 (21.8)	368 (50.2)	205 (28.0)	0.94 (0.7)	1 (1)
Not smoking	333 (45.4)	262 (35.7)	138 (18.8)	1.27 (0.75)	1 (1)
Using a hearing aid if you need one	128 (17.5)	289 (39.4)	316 (43.1)	0.74 (0.73)	1 (1)
Maintaining healthy blood pressure levels	254 (34.7)	365 (49.8)	114 (15.6)	1.19 (0.68)	1 (1)
Cutting down on alcohol if you drink excessively	462 (63.0)	180 (24.6)	91 (12.4)	1.51 (0.7)	2 (1)
Avoiding polluted air	279 (38.1)	307 (41.9)	147 (20.1)	1.18 (0.74)	1 (1)
Taking care to avoid head injury	329 (44.9)	290 (39.6)	114 (15.6)	1.29 (0.72)	1 (1)
Being active socially, such as doing volunteer work, going to discussion groups	265 (36.2)	286 (39.0)	182 (24.8)	1.11 (0.77)	1 (1)
Maintaining healthy blood sugar levels	282 (38.5)	339 (46.2)	112 (15.3)	1.23 (0.69)	1 (1)
Maintaining healthy cholesterol levels	288 (39.3)	327 (44.6)	118 (16.1)	1.23 (0.70)	1 (1)
Maintaining a healthy weight	286 (39.0)	327 (44.6)	120/(16.4)	1.23 (0.70)	1 (1)
Eating a Mediterranean diet (lots of fruit, vegetables, fish, grains, chickpeas and different types of beans and peas, and olive oil)	284 (38.7)	318 (43.4)	131 (17.9)	1.21 (0.72)	1 (1)
Getting help if you have depression	339 (46.2)	292 (39.8)	102 (13.9)	1.32 (0.7)	1 (1)

Misconceptions	Strongly disagree *n* (%)	Disagree *n* (%)	Strongly agree/ agree/neutral *n* (%)	Mean (SD) out of a maximum score of 2	Median (IQR) out of a maximum score of 2
Adding coconut oil to your diet	27 (3.7)	74 (10.1)	632 (86.2)	0.17 (0.46)	0 (0)
Avoiding artificial sweeteners	14 (1.9)	43 (5.9)	676 (92.2)	0.1 (0.35)	0 (0)
Taking nutritional supplements such as ginkgo	15 (2.0)	38 (5.2)	680 (92.8)	0.09 (0.35)	0 (0)
Using non-aluminum cookware	24 (3.3)	48 (6.5)	661 (90.2)	0.13 (0.42)	0 (0)
Having metal dental fillings removed	31 (4.2)	71 (9.7)	631 (86.1)	0.18 (0.48)	0 (0)
Eating a protein-rich diet (lots of meat, eggs)	15 (2.0)	32 (4.4)	686 (93.6)	0.08 (0.34)	0 (0)

**Table 4:** Preventative health actions and behaviors taken for dementia risk reduction (*n* = 733).

Actions and behaviors for dementia risk reduction	Yes, for dementia risk reduction *n* (%)	Yes, but not for dementia risk reduction *n* (%)	No *n* (%)	Prefer not to say *n* (%)
Check blood pressure by a doctor or other health professional at least every 2 years and maintain it within a healthy range	366 (49.9)	189 (25.8)	74 (10.1)	104 (14.2)
Check cholesterol level by a doctor or other health professional every 5 years and maintain it within a healthy range	326 (44.5)	234 (31.9)	70 (9.5)	103 (14.1)
Avoid smoking tobacco	377 (51.4)	227 (31.0)	39 (5.3)	90 (12.3)
Maintain a healthy weight	338 (46.1)	275 (37.5)	35 (4.8)	85 (11.6)
Manage depression (if applicable)	407 (55.5)	162 (22.1)	59 (8.0)	105 (14.3)
Doing regular physical activity (such as playing sport, brisk walking or gardening), engaging in at least 150 min of moderate intensity physical activity per week or at least 30 min of moderate physical activity on most days.	424 (57.8)	196 (26.7)	35 (4.8)	78 (10.6)
Check blood sugar levels by a doctor or other health professional every 3 years and maintain it within a healthy range	366 (49.9)	220 (30.0)	51 (7.0)	96 (13.1)
Keep socially active (such as doing volunteer work, going to discussion groups)	397 (54.2)	206 (28.1)	47 (6.4)	83 (11.3)
Avoid excessive alcohol consumption (more than 10 standard drinks a week and no more than 4 standard drinks on any 1 day).	360 (49.1)	145 (19.8)	29 (4.0)	65 (8.9)
Avoid sports that can cause head injury, or wear protective gear when you do	402 (54.8)	188 (25.6)	59 (8.0)	84 (11.5)
Follow the Mediterranean diet (lots of fruit, vegetables, fish, wholegrains, chickpeas and different types of beans and peas, and olive oil)	401 (54.7)	206 (28.1)	36 (4.9)	90 (12.3)
Keep cognitively active (such as participating in craft, hobbies, or similar leisure activities, playing games, participating in brain training activities, learning new music to play or sing, or undertaking study or taking a course)	424 (57.8)	188 (25.6)	38 (5.2)	83 (11.3)
Use a hearing aid (if you need one)	272 (37.1)	203 (27.7)	130 (17.7)	128 (17.5)
Avoid polluted air	312 (42.6)	281 (38.3)	43 (5.9)	97 (13.2)

**Table 5:** Stigma toward people living with dementia (PwD).

	SD^ *n* (%)	D^ *n* (%)	SWD^ *n* (%)	N^ *n* (%)	SWA^ *n* (%)	A^ *n* (%)	SA^ *n* (%)	Mean (SD) out of a maximum score of 7	Median (IQR) out of a maximum score of 7
Cognitive (dementia-related stereotypes)									
It is possible to enjoy interacting with PwD*	34 (4.6)	53 (7.2)	58 (7.9)	170 (23.2)	160 (21.8)	171 (23.3)	87 (11.9)	3.3 (1.6)	3 (2)
PwD can enjoy life*	36 (4.9)	58, (7.9)	77, (10.5)	166 (22.6)	152 (20.7)	160 (21.8)	84 (11.5)	3.4 (1.6)	3 (2)
PwD can feel when others are kind to them*	30 (4.1)	58 (7.9)	51 (7.0)	183 (25.0)	145 (19.8)	184 (25.1)	82 (11.2)	3.3 (1.6)	3 (2)
PwD are a burden to their family	115 (15.7)	95 (13.0)	69 (9.4)	176 (24.0)	150 (20.5)	98 (13.4)	30 (4.1)	3.8 (1.8)	4 (3)
PwD are a burden on the healthcare system	191 (26.1)	118 (16.1)	73 (10.0)	185 (25.2)	83 (11.3)	60 (8.2)	23 (3.1)	3.2 (1.76)	3 (3)
People PwD are very much like children	31 (4.2)	30 (4.1)	47 (6.4)	119 (16.2)	195 (26.6)	181 (24.7)	130 (17.7)	5.0 (1.6)	5 (2)
PwD are incapable of making any personal decisions	22 (3.0)	31 (4.2)	54 (7.4)	124 (16.9)	147 (20.1)	213 (29.1)	142 (19.4)	5.1 (1.5)	5 (2)
PwD are no longer themselves because they have dementia	31 (4.2)	31 (4.2)	50 (6.8)	151 (20.6)	155 (21.1)	191 (26.1)	124 (16.9)	5.0 (1.6)	5 (2)
PwD should always be supervised	23 (3.1)	19 (2.6)	31 (4.2)	92 (12.6)	105 (14.3)	232 (31.7)	231 (31.5)	5.5 (1.5)	6 (2)
PwD are unpredictable	69 (9.4)	69 (9.4)	80 (10.9)	240 (32.7)	124 (16.9)	97 (13.2)	54 (7.4)	4.1 (1.6)	4 (2)
Emotional (negative prejudices and emotional reactions)									
I am afraid of PwD	249 (34.0)	193 (26.3)	66 (9.0)	109 (14.9)	65 (8.9)	30 (4.1)	21 (2.9)	2.6 (1.7)	2 (3)
I feel confident around PwD*	68 (9.3)	118 (16.1)	112 (15.3)	241 (32.9)	98 (13.4)	67 (9.1)	29 (4.0)	4.3 (1.5)	4 (3)
I am comfortable touching PwD*	44 (6.0)	78 (10.6)	58 (7.9)	217 (29.6)	119 (16.2)	142 (19.4)	75 (10.2)	3.6 (1.7)	4 (2)
I feel relaxed around PwD*	34 (4.6)	68 (9.3)	81 (11.1)	252 (34.4)	130 (17.7)	118 (16.1)	50 (6.8)	3.7 (1.5)	4 (2)
Behavioral (discriminatory behaviors)									
I would ignore PwD	239 (32.6)	179 (24.4)	70 (9.5)	159 (21.7)	32 (4.4)	39 (5.3)	15 (2.0)	2.7 (1.62)	2 (3)
I would exclude PwD from activities	230 (31.4)	167 (22.8)	92 (12.6)	152 (20.7)	43 (5.9)	34 (4.6)	15 (2.0)	2.7 (1.6)	2 (3)

*Positively framed attitudes, reverse-scored to ensure that lower mean and median scores corresponded to lower levels of dementia-related stigma. ^ SD, Strongly disagree; D, Disagree, SWD, Somewhat disagree; N, Neither agree nor disagree; SWA, Somewhat agree; A, Agree; SA, Strongly agree.

### 2.5 Statistical analysis

Data analysis was conducted using IBM SPSS Statistics for Windows, Version 28 (IBM Corp., Armonk, NY). The total DKAS score was computed by summing the scores of all 25 items (range: 0 to 50). Each item was scored on a scale of 0 to 2 based on the following criteria: a score of 2 for a correct definitive answer (“True” for a true statement, “False” for a false statement), a score of 1 for a correct probable answer (“Probably True” or “Probably False”), a score of 0 for incorrect or “Did Not Know” responses. The scale demonstrated good reliability (Cronbach’s alpha = 0.85). Consistent with previous studies (16, 28, 29). A cutoff of 30 (the 60th percentile) was set to indicate sufficient knowledge. To facilitate descriptive comparisons and identify relative at-risk subgroups within the sample, the scores were subsequently categorized as low (0–29; < 60%), moderate (30–39), or high (≥40). These specific categorical bands are investigator-defined based on sample distribution rather than established population diagnostic thresholds.

For the 14 modifiable dementia risk factors (KoDeRR_R), responses were scored in accordance with the original validated KoDeRR methodology (40): strongly agree = 2, agree = 1, and all other responses = 0 (maximum score: 28). In the context of assessing health knowledge, a neutral response (“neither agree nor disagree”) indicates an absence of definitive correct knowledge, thus justifying its grouping with incorrect responses (score of 0). Total scores of knowledge levels were categorized into low (0–16; <60%), moderate (17–22), and high (≥ 23). For the six misconceptions (KoDeRR_M), reverse scoring was applied following the same established methodology (40): “Strongly Disagree” = 2, “Disagree” = 1, and other responses = 0 (maximum score: 12). Knowledge of misconceptions was categorized as low (0–7; <60%), moderate (8–9), or high (≥10). Summing these provided the overall KoDeRR score (range: 0–40), with higher total scores reflecting greater knowledge of dementia risk factors and better recognition of common misconceptions. Based on the 60% threshold, total scores were categorized into low (0–23; <60%), moderate (24–31), and high (≥32). The internal consistency was acceptable to excellent (Cronbach’s alphas: KoDeRR_R = 0.92; KoDeRR_M = 0.70; overall KoDeRR = 0.87).

The total DePSS score was calculated by summing the scores of all 16 items, including 6 reverse-scored positive items, so that lower scores indicated less stigma (range: 16–112). Also, the subscale scores were calculated to offer a more detailed understanding of stigma across its cognitive (10–70), emotional (4–28), and behavioral (2–14) dimensions. The total DePSS showed acceptable reliability (Cronbach’s alpha = 0.71). In the absence of established cutoffs for this scale, stigma levels were categorized using an investigator-defined, sample-based approach. For binary analyses, a median split was applied: <62 (low stigma), and ≥62 (high stigma). While median dichotomization compresses continuous variance, it was purposefully selected here to yield highly interpretable Adjusted Odds Ratios (AORs) for public health reporting. For the descriptive analysis, scores were classified into tertiles based on the 25th and 75th percentiles: low (≤55), moderate (56–68), and high (≥69). We explicitly note that these categorizations are sample-dependent and used for analytical purposes, not as diagnostic population thresholds.

Data normality was assessed using the Shapiro–Wilk and Kolmogorov–Smirnov tests. Because the continuous score data were non-normally distributed, results are reported as medians with interquartile ranges (IQRs), means and standard deviations (SDs) are included to allow for comparison with other literature. Categorical data are presented as percentages with 95% confidence intervals (CIs).

Univariable logistic regression analyses were initially performed to assess the associations between independent variables and the dependent outcomes. Based on established recommendations in the literature, variables with *p*-values < 0.25 in the univariable analyses were considered eligible for inclusion in the multivariable logistic regression models to account for potential confounding effects and improve model accuracy (42, 43). Additionally, theoretically important sociodemographic confounders were evaluated for inclusion. Prior to finalizing the multivariable models, the standard assumptions of logistic regression were evaluated. Specifically, multicollinearity was formally assessed using the Variance Inflation Factor (VIF), which confirmed the absence of problematic collinearity among predictors (all VIF values < 1.5). Formal interaction effects were not included, as no *a priori* interactions were hypothesized, and the primary objective was to identify independent main effects. Results are reported as unadjusted odds ratios (ORs) and adjusted odds ratios (AORs) with their respective 95% CIs. Statistical significance was set at *p* < 0.05, and model fit was assessed using the Hosmer–Lemeshow goodness-of-fit test.

For the regression analyses, the dependent variables were dichotomized as follows: knowledge (sufficient [moderate/high] = 1; low = 0) and stigma (low = 1; high = 0). All independent categorical variables were dummy-coded, using the first category as the reference. These predictors included age, gender, nationality, marital status, education, employment, residential area, monthly household income, working/studying in the medical field, personal experience with dementia, and professional experience with PwD. Spearman’s rank-order correlation (Spearman’s rho) was used to explore the relationships between: (1) general knowledge of dementia and the number of preventive actions or behaviors practiced; (2) modifiable risk factors knowledge and the number of preventive actions or behaviors practiced; (3) general knowledge and stigma; and (4) modifiable risk factors knowledge and stigma. Statistical significance for correlation analyses was also set at *p* < 0.05.

## 3 Results

### 3.1 Study population

A total of 733 participants completed the survey. Females comprised the majority of the sample (75.0%), as did Kuwaiti nationals (87.3%). Over one-third (36.0%) of respondents were aged between 18 and 24 years. A university-level education was prevalent: 33.0% were current undergraduates, and 51.4% held a university or postgraduate degree. In terms of occupation, 15.6% worked or studied within the medical field. Regarding dementia-related exposure, 48.2% personally knew someone with dementia (half of whom had served as caregivers), while 20.6% had professional experience working directly with PwD (Table 1).

### 3.2 General dementia knowledge

Assessment via the DKAS revealed an overall low level of general dementia knowledge across the sample. The median (IQR) score was 19.0 (12.0) out of a maximum 50, with a mean (SD) of 18.7 (9.0). A substantial majority of respondents (n = 648; 88.4%; 95% CI: 85.8–90.6) demonstrated a low level of knowledge. In contrast, 10.6% (n = 78; 95% CI: 8.6–13.2) exhibited moderate knowledge, while only 1.0% (n = 7; 95% CI: 0.4–2.1) achieved a high level of knowledge. Table 2 details responses to all 25 DKAS items.

Confidence in accurately identifying statements as definitively true or false was generally poor, highlighting widespread uncertainty about dementia-related knowledge. For instance, 65% of participants either mistakenly believed dementia is a normal part of aging or selected “Do not know,” with only 17.6% correctly recognizing the statement as false. A similar pattern emerged regarding the reversibility of the condition: 53.1% incorrectly believed it could be reversed or “Do not know,” whereas 17.2% correctly identified this as false. Misconceptions about specific subtypes and disease presentation were also common. A large majority (84.9%) either incorrectly identified vascular dementia as the most common type or did not know the correct answer. Furthermore, 87.3% erroneously believed or were unaware that the sudden onset of cognitive problems is typical of common forms of dementia.

### 3.3 Knowledge of dementia risk factors and misconceptions

Scores on the KoDeRR_R reflected low knowledge of modifiable risk factors, with a median (IQR) of 16.0 (9.0) and a mean (SD) of 16.5 (7.1) out of 28. Over half of the participants (*n* = 381; 52.0%; 95% CI: 48.3–55.6) showed low knowledge, while 23.6% (*n* = 173; 95% CI: 20.6–26.9) and 24.4% (*n* = 179; 95% CI: 21.4–27.7) demonstrated moderate and high knowledge, respectively. Understanding of common misconceptions (KoDeRR_M) was particularly poor. The median (IQR) score was 0 (1) out of 12, with a mean (SD) of 0.8 (1.6). Nearly all respondents (*n* = 727; 99.2%; 95% CI: 98.1–99.7) scored in the low range for this subdomain, with only a fraction achieving moderate (0.4%) or high (0.4%) scores. Given the conservative scoring methodology where neutral responses receive zero points, it is important to note that these exceptionally low scores reflect a combination of active endorsement of the misconceptions as well as widespread uncertainty.

Consequently, the combined KoDeRR score yielded a median (IQR) of 17.0 (10.0) and a mean (SD) of 17.2 (6.9) out of 40. Most respondents (*n* = 576; 78.6%; 95% CI: 75.4–81.5) exhibited an overall low level of knowledge regarding evidence-based modifiable risk factors and the identification of misconceptions, while 21.3% (*n* = 156; 95% CI: 18.4–24.5) had moderate knowledge, and 0.1% (*n* = 1; 95% CI: 0.01–0.9) had high knowledge. Notably, higher general dementia knowledge was positively correlated with knowledge of modifiable risk factors (Spearman’s rho = 0.333, *p* < 0.001).

Table 3 outlines participants’ responses to risk factor and misconception items. The majority correctly identified several modifiable risks, including reducing alcohol consumption (87.6%), seeking treatment for depression (86.0%), maintaining healthy levels of blood sugar (84.7%), blood pressure (84.5%), cholesterol (83.9%) and body weight (83.6%), avoiding head injuries (84.5%), adhering to a Mediterranean diet (82.0%), and avoiding smoking (81.1%). Awareness of protective behaviors was also evident for avoiding polluted air (80%), engaging in regular physical activity (77.3%), staying socially active (75.2%), and continuously learning new things (72%). Conversely, hearing aid use when needed was less recognized as a modifiable factor (56.9%). Even though many respondents accurately identified these factors, the general preference for “agree” over “strongly agree” indicates potential uncertainty or lack of confidence in their knowledge.

Recognition of false claims was markedly low. Less than one-fifth of respondents correctly categorized misconceptions by selecting “disagree” or “strongly disagree,” and softer disagreement was far more common, suggesting a potential lack of confidence in their knowledge. Because neutral or unsure responses were scored as zero alongside incorrect answers, these low recognition rates highlight a broader lack of definitive knowledge rather than exclusively firm belief in these myths. Only 13.9 and 13.8% accurately dismissed the removal of metal dental fillings or the addition of coconut oil to the diet as valid preventive strategies. Awareness was even lower for other commonly held misconceptions, including using non-aluminum cookware (9.8%), avoiding artificial sweeteners (7.8%), taking nutritional supplements such as *ginkgo biloba* (7.2%), and following a high-protein diet (6.4%) as strategies for dementia prevention.

### 3.4 Preventative health actions or behaviors taken for dementia risk reduction

Participants reported engaging in a median (IQR) of 7.0 (11.0) out of 14 possible risk-reducing behaviors, with a mean (SD) of 7.2 (5.2). Statistical analysis revealed that greater dementia knowledge was significantly associated with reporting a higher number of risk-reducing behaviors. Significant positive correlations linked the number of actions taken with both general dementia knowledge (Spearman’s rho = 0.262, *p* < 0.001) and knowledge of modifiable risk factors (Spearman’s rho = 0.258, *p* < 0.001).

As detailed in Table 4, the behaviors most frequently adopted with the explicit goal of reducing dementia risk included staying physically and cognitively active (57.8%), managing depression (55.5%), avoiding sports associated with head injury risk (54.8%), following a Mediterranean diet (54.7%), and maintaining social engagement (54.2%). Conversely, avoiding air pollution (42.6%) and using necessary hearing aids (37.1%) were less commonly practiced for this purpose. More than a quarter of participants engaged in up to 12 health-promoting actions or behaviors, though not necessarily adopted with the explicit intention of preventing dementia.

Accessibility of preventive information appears limited, as only 30.7% of participants (*n* = 225; 95% CI: 27.4–34.2) found materials on dementia risk reduction easy to obtain. Furthermore, 63.7% (*n* = 467; 95% CI: 60.1–67.2) indicated they would be more likely to adopt lifestyle changes if they knew such changes could lower their risk of developing dementia.

### 3.5 Stigma toward people living with dementia

Public stigma toward dementia was generally moderate. The total DePSS score showed a median (IQR) of 62.0 (13.0) out of 112, with a mean (SD) of 61.3 (11.2). While 27.2% of participants (*n* = 199; 95% CI: 24.0–30.6) displayed low stigma, nearly half (*n* = 355; 48.4%; 95% CI: 44.8–52.1) demonstrated moderate stigma, and 24.4% (*n* = 179; 95% CI: 21.4–27.7) exhibited high stigma. Sub-dimension analysis revealed moderate stigma within the cognitive [median (IQR) = 41.0 (9.5)/70 and a mean (SD) = 41.7 (7.8)] and emotional [median (IQR) = 15.0 (6.0)/28 and a mean (SD) = 14.3 (4.2)] domains. The behavioral dimension, however, reflected lower stigma levels [median (IQR) = 4.0 (6.0)/14 and a mean (SD) of 5.3 (3.0)]. Respondents with higher levels of dementia knowledge demonstrated significantly lower levels of dementia-related stigma. This was evidenced by inverse correlations between stigma and both general dementia knowledge (Spearman’s rho = −0.10, *p* = 0.012) and risk factors awareness (Spearman’s rho = −0.12, *p* = 0.001).

Table 5 outlines responses to specific attitude statements. For the 10 negatively framed items, disagreement (i.e., somewhat disagree, disagree, or strongly disagree) ranged from 9.9 to 69.0%, with neutral responses spanning 12.6 to 32.7%. For the six positively framed items, levels of agreement (somewhat agree, agree, or strongly agree) ranged from 26.5 to 57.0%, with neutral responses between 22.6 and 34.4%.

Negative attitudes were prevalent among more than 60% of respondents. High proportions agreed that PwD should always be supervised (77.5%), behave like children (69.0%), are incapable of making personal decisions (68.6%), and are no longer themselves (64.1%). However, fewer viewed PwD as a burden on the healthcare system (22.6%), or their families (38.0%), or considered them unpredictable (37.5%).

Positive attitudes were also evident. Over half of the respondents agreed that PwD can enjoy life (54.0%), that interacting with them can be enjoyable (57.0%), and that they can perceive others’ kindness (56.1%). Emotionally, 69.3% reported not being afraid of PwD, 45.8% felt comfortable touching them, 40.6% felt relaxed around them, and 26.5% felt confident around them. Behaviorally, a small minority indicated that they would exclude PwD from activities (12.5%) or ignore them (11.7%).

### 3.6 Help-seeking behaviors

Respondents’ help-seeking behaviors were explored, emphasizing their importance in facilitating early diagnosis and reducing the stigma associated with receiving or disclosing a dementia diagnosis. Among the 529 participants (72.2%) who specified whom they would inform about a personal dementia diagnosis, the majority (n = 490; 92.6%; 95% CI: 90.0–94.6) selected family and friends. Disclosure to employers, colleagues (n = 24; 4.5%), or neighbors (*n* = 15; 2.8%) was rarely chosen. The remaining participants were either uncertain or preferred not to disclose this information at all.

Overall, 66.7% (*n* = 489; 95% CI: 63.2–70.1) expressed willingness to seek help upon experiencing symptoms of dementia. In contrast, 6.8% (*n* = 50) stated they would avoid seeking help, 17.5% (*n* = 128) remained unsure, and 9.0% (*n* = 66) preferred not to say. Of those willing to seek help, 50.7% (n = 248; 95% CI: 46.2–55.2) indicated they would do so immediately. Others would delay action, either waiting a while (*n* = 165; 33.7%; 95% CI: 29.6–38.2) or waiting until symptoms became apparent to others (*n* = 76; 15.5%; 95% CI: 12.5–19.1).

Among the 656 respondents (89.5%) who indicated where they would seek help for dementia symptoms, the most commonly preferred source was medical specialists (*n* = 339; 51.7%; 95% CI: 47.8–55.6), followed by family and friends (*n* = 158; 24.1%; 95% CI: 20.9–27.6), general practitioners (*n* = 119; 18.1%; 95% CI: 15.3–21.4), and online sources (*n* = 40; 6.1%; 95% CI: 4.4–8.3).

### 3.7 Univariable and multivariable logistic regression analyses

#### 3.7.1 General dementia knowledge

Univariable logistic regression identified marital status, medical field affiliation, and professional experience with PwD as factors significantly associated with general dementia knowledge. Married participants had lower knowledge compared to single/divorced/widowed individuals (OR = 0.53; 95% CI: 0.33–0.85; *p* = 0.008). Working or studying in the medical field was strongly associated with higher knowledge (OR = 9.44; 95% CI: 5.76–15.47; *p* < 0.001), as did professional experience with PwD (OR = 2.38; 95% CI: 1.46–3.87; *p* < 0.001). Nationality (OR = 1.57; 95% CI: 0.85–2.87; *p* = 0.147), monthly income (500–1,000 KD: OR = 0.72; 95% CI: 0.36–1.43; >1,000 KD: OR = 1.39; 95% CI: 0.84–2.29; *p* = 0.112), and personal experience with PwD (OR = 1.46; 95% CI: 0.93–2.30; *p* = 0.104) did not reach statistical significance. Other remaining variables were excluded from the multivariable model based on the inclusion criteria (*p* < 0.25).

In the multivariable analysis, marital status remained significantly associated with lower knowledge (AOR = 0.47; 95% CI: 0.26–0.85; *p* = 0.012). Both medical field affiliation (AOR = 8.53; 95% CI: 5.11–14.23, *p* < 0.001) and professional experience with PwD (AOR = 2.45; 95% CI: 1.36–4.42; *p* = 0.003) retained their strong positive associations. The adjusted model confirmed no significant associations for non-Kuwaiti nationality (AOR = 1.81; 95% CI: 0.90–3.62; *p* = 0.094), personal experience with PwD (AOR = 1.21; 95% CI: 0.70–2.09; *p* = 0.506), or income (500–1,000 KD: AOR = 1.01; 95% CI: 0.46–2.21; >1,000 KD: AOR = 1.56, 95% CI: 0.86–2.83, *p* = 0.259). The model exhibited a good fit (Hosmer–Lemeshow test: *p* = 0.995).

#### 3.7.2 Overall knowledge of dementia risk factors and misconceptions

Univariable analysis indicated that married participants possessed lower overall KoDeRR (OR = 0.67; 95% CI: 0.47–0.96; *p* = 0.030). Working or studying in the medical field showed a non-significant association (OR = 1.54; 95% CI: 0.98–2.43; *p* = 0.061). Other variables were excluded based on the inclusion criteria (*p* < 0.25). In the multivariable model, neither marital status (AOR = 0.70; 95% CI: 0.49–1.00; *p* = 0.053) nor medical field affiliation (AOR = 1.45; 95% CI: 0.92–2.29; *p* = 0.114) were statistically significant. The goodness-of-fit for this model was acceptable (Hosmer–Lemeshow test: *p* = 0.555).

#### 3.7.3 Stigma toward PwD

In the univariable analysis, professional experience with PwD significantly predicted lower stigma (OR = 2.06; 95% CI: 1.42–2.98; *p* < 0.001). Neither working or studying in the medical field (OR = 1.31; 95% CI: 0.88–1.96; *p* = 0.183) nor personal experience with PwD (OR = 1.28; 95% CI: 0.96–1.71; *p* = 0.098) demonstrated significant associations. Remaining independent variables were excluded based on the inclusion criteria (*p* < 0.25). The multivariable analysis confirmed that professional experience with PwD was the sole significant independent factor associated with lower stigma (AOR = 2.04; 95% CI: 1.36–3.07; *p* < 0.001). Other predictors remained statistically insignificant: working or studying in the medical field (AOR = 1.29; 95% CI: 0.86–1.93; *p* = 0.232), and personal experience with PwD (AOR = 1.01; 95% CI: 0.73–1.39; *p* = 0.978). The model demonstrated a strong fit (Hosmer–Lemeshow test: *p* = 0.982).

## 4 Discussion

To the best of our knowledge, this study represents the first comprehensive assessment in Kuwait and the broader Middle East and only the second globally following the Australian national survey (23) to simultaneously evaluate public dementia knowledge, awareness of modifiable risk factors, belief in misconceptions, and stigma toward PwD. Previous studies in Kuwait had a narrower focus, targeting university students using an earlier version of the DKAS (35) or healthcare professionals (36) without evaluating awareness of risk factors and misconceptions or stigma. Similarly, studies from neighboring countries, such as Saudi Arabia (29) investigated isolated aspects of dementia knowledge or focused specifically on public and healthcare students’ knowledge and attitudes toward AD (30–34). By integrating these diverse constructs into a single population-wide analysis, the current study contributes valuable insights to the regional literature on dementia literacy and public stigma and provides a critical baseline for developing targeted regional health policies.

The present findings reveal suboptimal general dementia knowledge in Kuwait, reflected by a mean DKAS score of 18.7 out of 50. This aligns closely with previous findings of Kuwaiti healthcare professionals (18.99) (36) and lower than general population scores in Saudi Arabia (20.5) (29) and Australia (21.0) (23) though slightly above Poland (16.95) (26). The relatively low score emphasizes the need for national public health initiatives to improve dementia literacy in Kuwait. The persistence of low baseline knowledge across both the general public and local healthcare professionals indicates a systemic gap in dementia education within the region that requires immediate national intervention.

A particularly concerning pattern of misinformation emerged regarding the etiology and trajectory of dementia. Sixty-five percent of participants incorrectly viewed dementia as a normal part of aging, a rate significantly higher than in Australia (27%) (23) and Poland (44.7%) (26), though comparable to Saudi Arabia (60%) (29). Furthermore, over half (53.1%) mistakenly believed or were unsure whether dementia could be cured. Holding these dual beliefs that cognitive decline is both inevitable and reversible creates a dangerous paradox that severely discourages early help-seeking. If individuals assume symptoms are an ordinary part of growing older or easily cured later, they are unlikely to prioritize early diagnostic evaluations. Evidence suggests that correcting such misconceptions could facilitate timely diagnosis, reduce stigma, and improve care outcomes. Public education must explicitly clarify that while age is a risk factor, neurological degradation is a disease process, and while incurable, early intervention is vital for managing disease progression.

Interestingly, 83% of participants successfully identified AD as the most common form of dementia, higher than recognition rates reported in Saudi Arabia (75%) (29), Poland (67.1%) (26), and Australia (63%) (23). This may reflect greater public exposure to AD-related messaging through health campaigns or media in Kuwait that frequently use AD as a catch-all term for cognitive decline. However, recognizing the disease name did not translate into a functional understanding of prevention or management, suggesting the need for more comprehensive public education. Nearly half of the respondents failed to recognize the protective role of a healthy lifestyle, echoing similar deficits in Saudi Arabia (29), Ireland (24), Norway (25), and Germany (27). This discrepancy points to a possible flaw in current health communication: campaigns may have successfully popularized dementia terminology but appear to have largely failed to communicate its modifiable nature. This underscores the need for public education campaigns that emphasize dementia prevention through lifestyle modifications, intending to promote proactive health behaviors that reduce the risk of developing dementia.

Analysis of the KoDeRR scales revealed a complex relationship with risk factor awareness. Participants demonstrated relatively high recognition of established risks like alcohol consumption, managing depression, not smoking, avoiding head injuries, and maintaining healthy blood sugar, blood pressure, and cholesterol levels, scoring higher on average than Australian cohorts (23). Yet, this apparent awareness is contradicted by a profound susceptibility to pseudoscientific myths. A majority failed to correctly reject claims such as a protein-rich diet (93.6%), avoiding artificial sweeteners (92.2%), and using non-aluminum cookware (90.2%) as preventive strategies. However, as reflected by the conservative scoring distribution, this poor performance likely indicates not only a firm belief in these myths but also a high degree of uncertainty, highlighting a general lack of confidence in distinguishing evidence-based facts from popular fiction. As such, the coexistence of relatively high agreement with established risk factors and frequent endorsement of unsupported preventive strategies may suggest an overall difficulty in distinguishing evidence-based recommendations from misinformation. It may also reflect a general tendency toward agreement with health-related statements, rather than a strictly evidence-based understanding of neurological health. Consequently, future public health messaging in Kuwait must combat pseudoscientific beliefs and public uncertainty through evidence-based public health communication that helps the public focus on modifiable risk factors with proven benefits for dementia prevention.

Regarding preventive behaviors, participants in Kuwait reported engaging in an average of 7.2 risk-reducing actions, notably higher than the 1.5 average reported in Australia (23). Frequently cited behaviors included maintaining physical activity and following a Mediterranean diet. The high engagement in these specific behaviors is promising, though we hypothesize this may be driven by broader metabolic and cardiovascular health campaigns (such as diabetes prevention, which is heavily promoted in the Gulf region) rather than targeted dementia awareness. However, behaviors such as avoiding air pollution and using hearing aids were reported less frequently. This suggests that specific dementia-linked actions, especially those related to sensory and environmental risks, require further attention in health promotion efforts, as both are linked to cognitive decline.

Multivariable modeling identified marital status, medical affiliation, and professional experience with PwD as primary factors associated with general knowledge. While age is frequently reported as an associated factor in international literature, it did not reach statistical significance in our cohort. Instead, marital status (being married) emerged as an independent factor associated with lower knowledge. This unexpected association must be interpreted with caution. Given our sample’s demographics, where over a third of participants were young adults aged 18–24 years, being married within this specific population may act as a proxy for other unmeasured life demands, such as specific caregiving responsibilities or family obligations, which could limit time for health information exposure. Because of the cross-sectional design of this study, this finding represents an association rather than a causal relationship. Unlike studies in Australia and Europe, where education, gender, and income are strong protective determinants against ignorance (23–25, 28), the current findings indicate that knowledge deficits regarding modifiable risks and misconceptions are pervasive across all socioeconomic and demographic strata in Kuwait. As such, public health education efforts should adopt a broad, population-wide approach rather than targeting only specific groups. Notably, although participants in the medical field demonstrated higher general knowledge of dementia, their awareness of risk factors remained limited. This finding underscores the importance of integrating dementia prevention education more robustly into healthcare curricula and continuing professional development programs. Given their trusted role in health communication, healthcare professionals must be well-equipped to convey accurate, evidence-based information and to support dementia risk reduction strategies in the wider community.

Stigma toward dementia remains a substantial challenge. The moderate overall DePSS scores in this study exceed those reported in Australia **[23]**, characterized heavily by cognitive and emotional dimensions. Although behavioral stigma appeared low, it is highly susceptible to social desirability bias; cultural and religious norms in the Middle East strongly emphasize respecting older adults, which we hypothesize may suppress the reporting of overtly discriminatory behaviors. Instead, the data reveals a complex profile of paternalistic attitudes, or ‘benevolent stigma.’ The coexistence of a willingness to interact with PwD alongside strong beliefs that they behave like children, cannot make decisions, and require constant supervision suggests that while PwD are treated with compassion, they are systematically stripped of their adult autonomy. Crucially, higher dementia knowledge and professional experience with PwD correlated with lower stigma levels. However, given the relatively weak effect sizes of these observed correlations, increasing factual knowledge alone may not be sufficient to produce substantial reductions in stigma. This suggests that comprehensive educational interventions must go beyond simply providing information and be specifically designed to actively foster empathy and deeper understanding toward PwD to effectively reduce stigma.

Despite reasonable awareness of the benefits of early diagnosis, only 66.7% of participants stated they would seek medical help if they experienced symptoms, significantly lower than the 94% reported in Australia (23). This indicates some uncertainty and hesitation among respondents. While 50.7% stated they would seek help immediately, this contrasts with 62.1% who responded “False” or “Probably False” to the statement that early diagnosis does not generally improve quality of life (Table 2). This gap between knowledge and action may reflect internalized stigma or fear of labeling, suggesting a need for health campaigns that reduce self-stigma and promote early healthcare-seeking behavior.

### 4.1 Limitations

The interpretation of the findings should be considered in light of the following potential limitations. First, selection bias may have occurred due to the use of a non-probability online snowball sampling strategy, which likely restricted participation to individuals with internet access, higher digital literacy, and potentially a greater pre-existing interest in health-related topics. Additionally, the overrepresentation of females, Kuwaiti nationals, and participants with university-level education further limits the generalizability of the findings to the broader population. Second, response and recall bias are possible, as the data were self-reported; participants may have overestimated socially desirable behaviors or underreported undesirable ones. Although anonymity may have reduced bias, the accuracy of responses could not be verified. Third, non-response bias cannot be excluded as individuals who declined to participate may have possessed different levels of knowledge or stigma toward dementia, potentially influencing the representativeness of the findings. Finally, the cross-sectional design of the study captures data at a single point in time, which limits the ability to assess changes in dementia knowledge and stigma over time and precludes the establishment of causal relationships. Furthermore, while our multivariable analyses adjusted for several key sociodemographic variables, the potential influence of unmeasured confounders, such as specific caregiving responsibilities or family obligations, cannot be entirely ruled out.

## 5 Conclusion

This study provides the first comprehensive investigation in the Middle East and only the second globally, preceded only by the Australian national survey (23), to simultaneously evaluate public dementia literacy, risk factors awareness, endorsement of misconceptions, and societal stigma within a single population framework. The findings reveal significant gaps in knowledge, widespread misconceptions about risk reduction, and limited awareness of evidence-based preventive measures. Despite recognizing certain lifestyle risks, the Kuwaiti population harbors deep-seated misconceptions regarding both dementia prevention and its etiology. Stigmatizing attitudes remain prevalent, particularly the negative stereotypes concerning the independence and cognitive abilities of PwD. Importantly, higher levels of dementia knowledge were associated with lower stigma, highlighting the role of public education in shaping attitudes.

These findings underscore the urgent need for comprehensive public health initiatives to enhance dementia literacy, correct misconceptions, and promote evidence-based prevention strategies. Given that knowledge gaps and stigmatizing beliefs were not confined to specific demographic groups, these efforts should target the general population. Integrating dementia awareness programs into national health policies, particularly within community and healthcare settings, may support earlier help-seeking, reduce stigma, and foster preventive health behaviors. Additionally, enhancing dementia-related content in medical and allied health curricula could better equip healthcare professionals to serve as trusted sources of information and advocates for dementia prevention.

Future research should evaluate the effectiveness of culturally tailored educational interventions and further examine the role of healthcare providers in improving dementia awareness and influencing public attitudes. These efforts are essential in the context of Kuwait’s aging population and growing dementia burden and can inform broader regional and global strategies to promote brain health and dementia prevention.

## Data Availability

The original contributions presented in the study are included in the article/supplementary material, further inquiries can be directed to the corresponding author/s.
